# The Diagnostic Mirage: Xanthogranulomatous Cholecystitis and the Illusion of Gallbladder Carcinoma

**DOI:** 10.7759/cureus.114571

**Published:** 2026-08-15

**Authors:** Anusha Prasad K, Akhila K, Sharan Basavaraj Javali

**Affiliations:** 1 General Surgery, Vydehi Institute of Medical Sciences and Research Centre, Bangalore, IND; 2 General Surgery, Vydehi Institute of Medical Sciences and Research Center, Bangalore, IND

**Keywords:** computed tomography, differential diagnosis, frozen section, gallbladder carcinoma, gallbladder wall thickening, histopathology, laparoscopic cholecystectomy, magnetic resonance cholangiopancreatography, radical cholecystectomy, xanthogranulomatous cholecystitis

## Abstract

Xanthogranulomatous cholecystitis (XGC) is an uncommon chronic inflammatory disease of the gallbladder that frequently mimics gallbladder carcinoma because of its overlapping clinical, radiological, and intraoperative features. Establishing a definitive preoperative diagnosis remains challenging, and intraoperative frozen section examination can aid surgical decision-making in patients with indeterminate gallbladder lesions while avoiding unnecessary radical resection. We present a case of a 49-year-old man with poorly controlled type 2 diabetes mellitus who presented with intermittent upper abdominal pain, low-grade fever, generalised pruritus, and insignificant weight loss. Initial ultrasonography demonstrated focal wall thickening involving the fundus and body of the gallbladder without definite sonographic features suggestive of malignancy. To rule out other intra-abdominal causes of his symptoms, contrast-enhanced computed tomography was performed, followed by magnetic resonance cholangiopancreatography (MRCP), which demonstrated focal asymmetric wall thickening involving the fundus and distal body of the gallbladder with diffusion restriction, raising suspicion for gallbladder carcinoma. Routine biochemical investigations were largely unremarkable except for elevated alkaline phosphatase, while serum tumour markers (CEA (carcinoembryonic antigen), CA 19-9 (carbohydrate antigen 19-9), and AFP (alpha-fetoprotein) ) were within normal limits. The patient underwent laparoscopic cholecystectomy with intraoperative frozen section examination, which excluded malignancy and allowed completion of the planned procedure without radical resection. Definitive histopathological examination confirmed XGC without evidence of dysplasia or malignancy. The postoperative recovery was uneventful, and the patient was discharged in stable condition. This case highlights the diagnostic challenge of differentiating XGC from gallbladder carcinoma despite contemporary imaging techniques. A systematic approach integrating sequential imaging, selective intraoperative frozen section examination, and definitive histopathological evaluation is essential for establishing the correct diagnosis and preventing unnecessary radical surgery in patients with indeterminate gallbladder lesions.

## Introduction

Xanthogranulomatous cholecystitis (XGC) is an uncommon destructive variant of chronic cholecystitis characterised by severe fibro-inflammatory changes and accumulation of lipid-laden macrophages within the gallbladder wall. Although benign in nature, it represents an important diagnostic challenge because its clinical, radiological, and intraoperative features frequently resemble those of gallbladder carcinoma, making definitive preoperative differentiation difficult [1]. The exact pathogenesis of XGC remains incompletely understood; however, chronic cystic duct obstruction and rupture of the Rokitansky-Aschoff sinuses are considered pivotal events that result in bile extravasation into the gallbladder wall and trigger an intense xanthogranulomatous inflammatory response. Progressive inflammation leads to marked mural thickening, fibrosis, xanthogranulomatous nodules, and dense adhesions to adjacent structures, producing imaging findings that closely mimic locally advanced gallbladder carcinoma [2]. Despite advances in ultrasonography, contrast-enhanced computed tomography (CECT), and magnetic resonance imaging, distinguishing XGC from gallbladder carcinoma remains challenging because both conditions commonly demonstrate focal or diffuse gallbladder wall thickening, heterogeneous enhancement, diffusion restriction, loss of tissue planes, and surrounding inflammatory changes. Consequently, no single radiological feature reliably excludes malignancy, and imaging findings should always be interpreted in conjunction with operative and pathological assessment [3]. When preoperative investigations remain inconclusive, intraoperative frozen section examination serves as a valuable adjunct for real-time differentiation between benign inflammatory disease and gallbladder carcinoma. Histological assessment during surgery can guide the extent of resection, allowing completion of an appropriate surgical procedure while avoiding unnecessary radical surgery in patients without malignancy. Nevertheless, definitive histopathological examination remains the gold standard for establishing the diagnosis [4]. 

The present report describes a patient with poorly controlled type 2 diabetes mellitus who presented with upper abdominal pain, constitutional symptoms, and progressive radiological findings that raised suspicion for gallbladder carcinoma despite negative tumour markers. Diagnostic uncertainty was resolved through intraoperative frozen section examination, while the final diagnosis of XGC was confirmed on definitive histopathological examination. This case highlights the importance of integrating sequential diagnostic imaging, selective intraoperative frozen section examination, and histopathological evaluation to accurately differentiate XGC from gallbladder carcinoma and optimise surgical decision-making.

## Case presentation

A 49-year-old man presented to the General Surgery outpatient department with intermittent upper abdominal pain of one and a half months' duration. The pain was dull aching, localised to the epigastrium and right upper quadrant, non-radiating, and partially relieved with analgesics. It was associated with intermittent low-grade fever, which was relieved on antipyretics, generalised pruritus, and insignificant weight loss. There was no history of nausea, vomiting, anorexia, altered bowel or bladder habits, or previous similar episodes. The patient was a known case of poorly controlled type 2 diabetes mellitus for five years and was receiving oral hypoglycaemic therapy with metformin, glimepiride, linagliptin, and empagliflozin. He had a smoking history of approximately 10-14 pack-years and had quit one year before presentation. There was no history of hypertension, bronchial asthma, tuberculosis, previous abdominal surgery, drug allergy, or a significant family history of malignancy. On examination, the patient was conscious, cooperative, and well oriented to time, place, and person. He was moderately built and nourished. Mild icterus was present, while pallor, clubbing, cyanosis, lymphadenopathy, and pedal oedema were absent. His blood pressure was 126/84 mmHg, pulse rate was 88 beats/minute, respiratory rate was 18 breaths/minute, temperature was 37°C, and oxygen saturation on room air was 99%. Abdominal examination revealed localised tenderness over the epigastrium and right hypochondrium without guarding, rigidity, organomegaly, palpable mass, or ascites. The remainder of the systemic examination was unremarkable.

Routine laboratory investigations were largely within normal limits, except for elevated alkaline phosphatase levels. Serum carcinoembryonic antigen (CEA) and carbohydrate antigen 19-9 (CA 19-9) were within normal limits, reducing the likelihood of gallbladder malignancy despite the patient’s persistent symptoms of abdominal pain (Table 1). 

**Table 1:** Clinically relevant laboratory investigations AST/ALT: Aspartate Aminotransferase/Alanine Aminotransferase, HbA1c: Glycated Hemoglobin, CEA: carcinoembryonic antigen, CA 19-9: carbohydrate antigen 19-9, HBsAg: hepatitis B surface antigen

Parameter	Result	Clinical interpretation
Haemoglobin	12.0 g/dL	Within acceptable limits
Total leucocyte count	5.81 × 10⁹/L	No leukocytosis
Total bilirubin	1.06 mg/dL	Within normal limits
Direct bilirubin	0.72 mg/dL	Mild elevation
AST/ALT	59/68 U/L	Mild transaminitis
Alkaline phosphatase	427 U/L	Elevated
Serum creatinine	0.80 mg/dL	Normal renal function
HbA1c	7.6%	Poorly controlled diabetes mellitus
CEA	Within normal limits	No biochemical evidence suggestive of malignancy
CA 19-9	Within normal limits	No biochemical evidence suggestive of malignancy
Alpha-fetoprotein (AFP)	Within normal limits	No biochemical evidence suggestive of malignancy
HBsAg	Positive	Chronic hepatitis B infection

**Figure 1:**
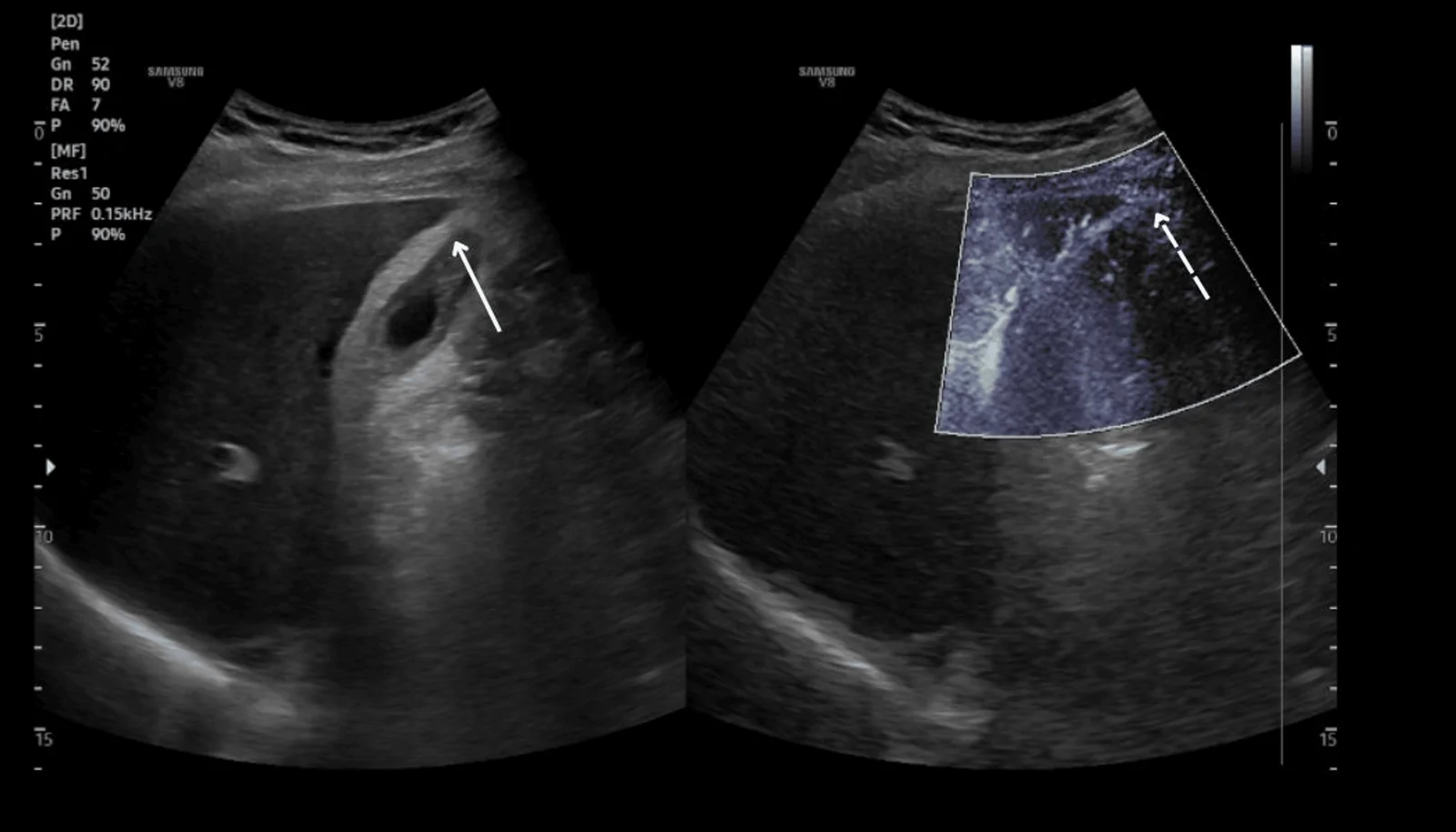
B-mode ultrasound image (curvilinear probe) showing focal wall thickening (left) and microvascular flow showing no increase in vascularity (right) Left: arrowhead pointing toward focal wall thickening at the fundus of the gallbladder. Right: dotted arrowhead pointing toward the fundus of the gallbladder, showing no increase in vascularity.

Initial ultrasonography demonstrated focal wall thickening of the gallbladder fundus and body with inflammatory hyperemia, associated with cholelithiasis and an echogenic intraluminal lesion, without definite imaging features suggestive of malignancy (Figure 1). 

**Figure 2:**
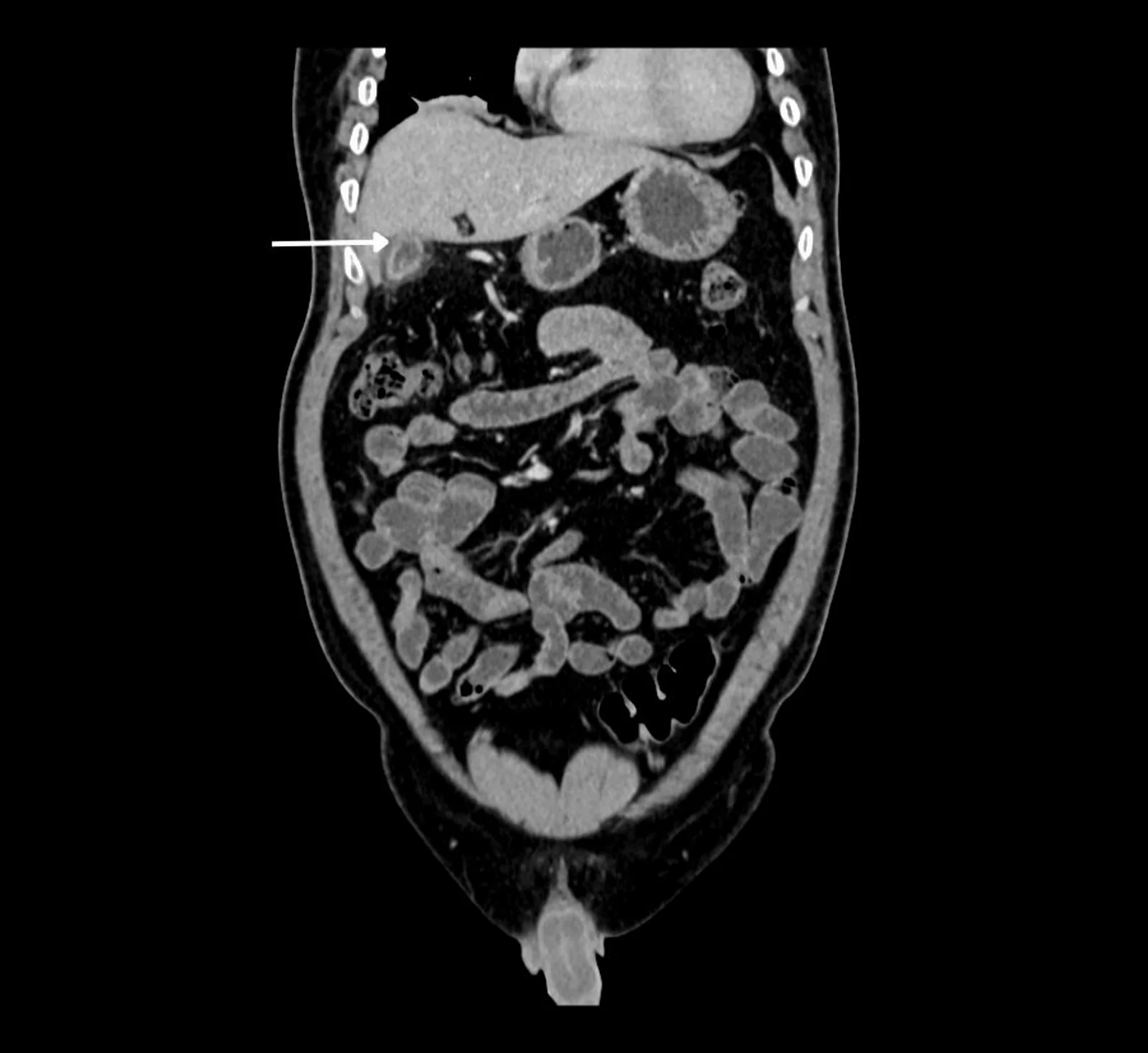
Coronal section of the contrast-enhanced computed tomography (CECT) abdomen showing a thick-walled gallbladder with cholelithiasis The arrowhead points to the gallbladder wall thickening with cholelithiasis.

**Figure 3:**
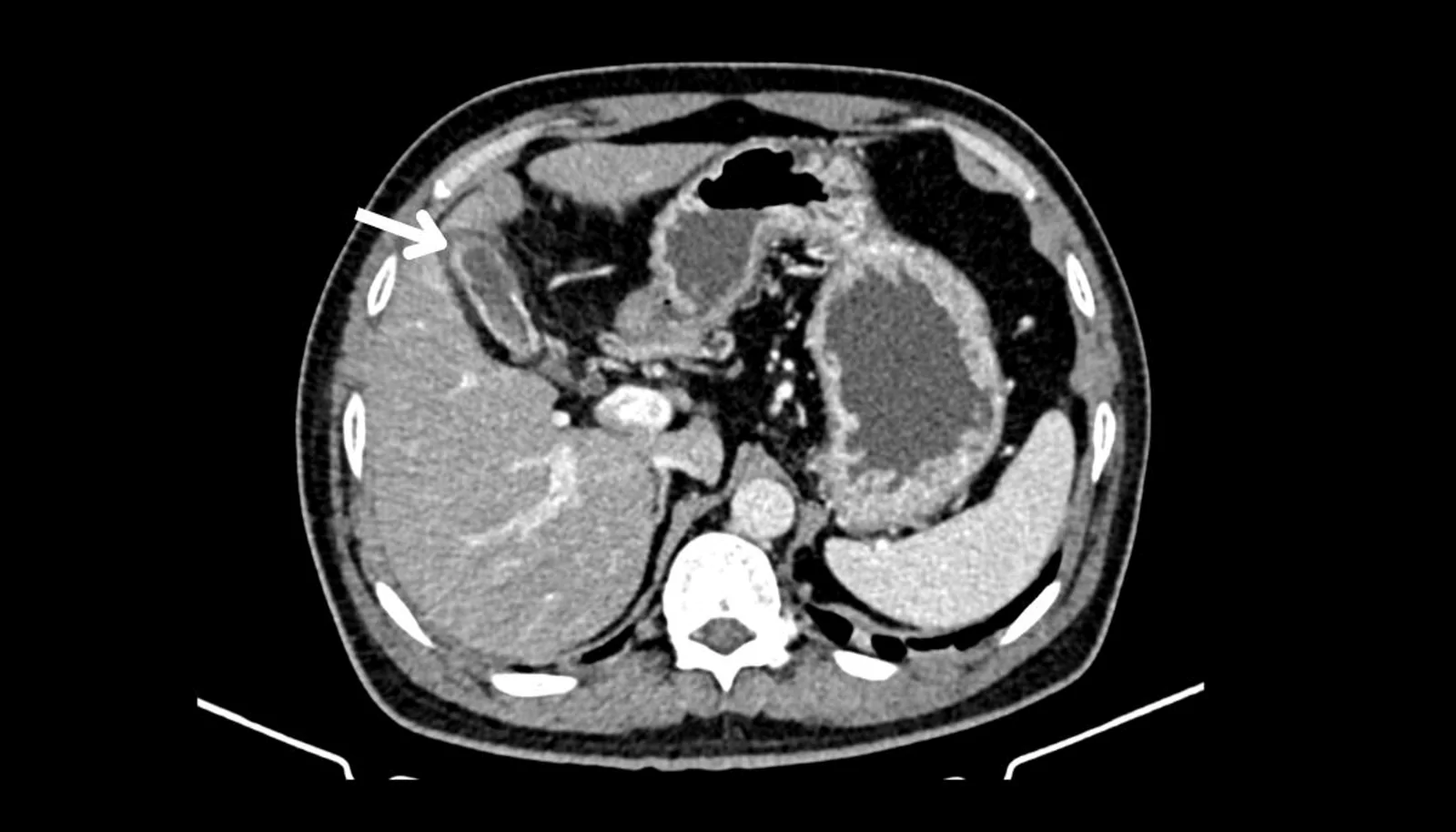
Axial section of the contrast-enhanced computed tomography (CECT) abdomen showing a thick-walled gallbladder with cholelithiasis The arrowhead points to the thickened gallbladder wall.

**Figure 4:**
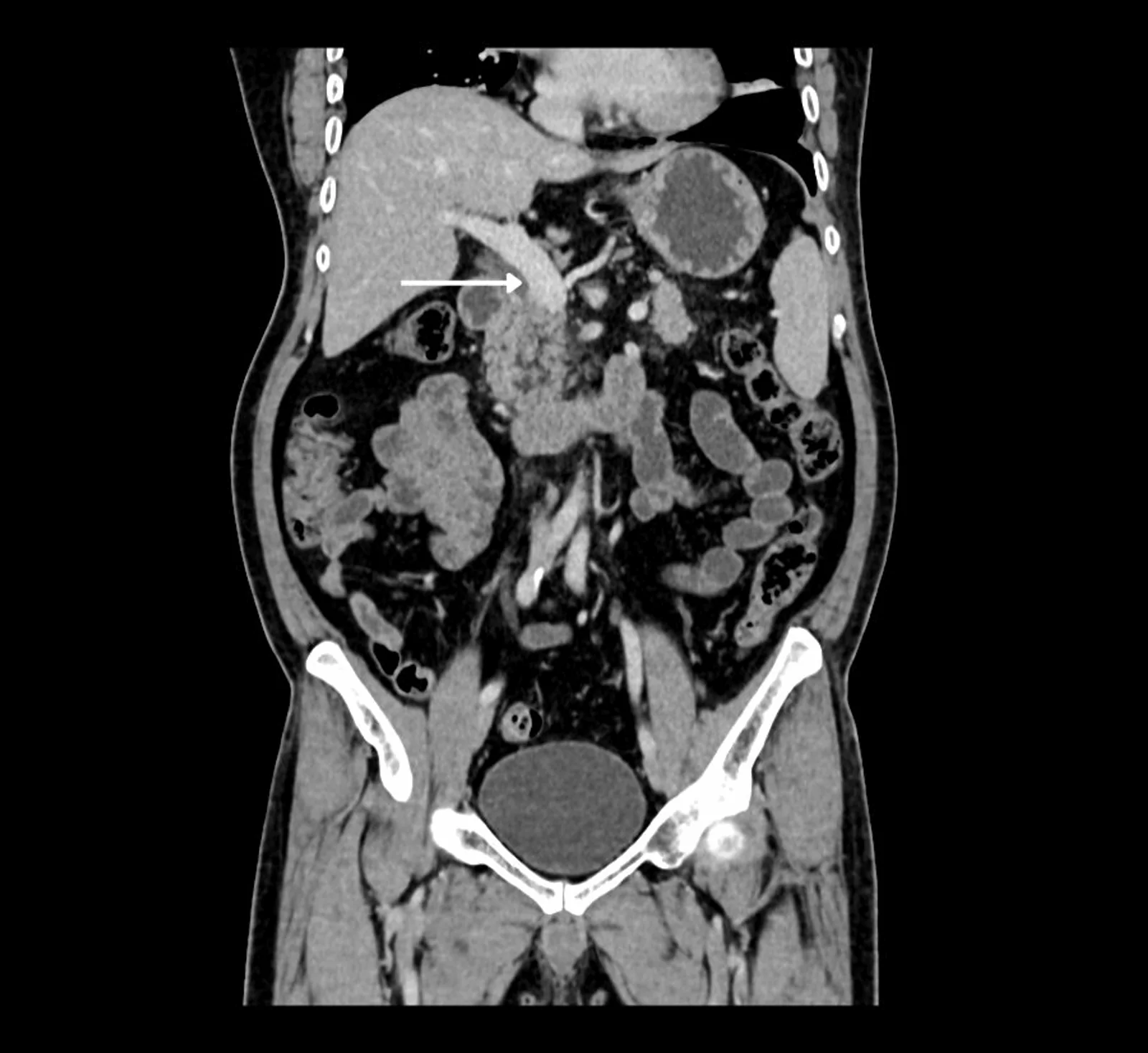
Coronal section of the contrast-enhanced computed tomography (CECT) abdomen showing dilatation of the proximal and distal common bile duct (CBD) The arrowhead points to the distal common bile duct (CBD).

**Figure 5:**
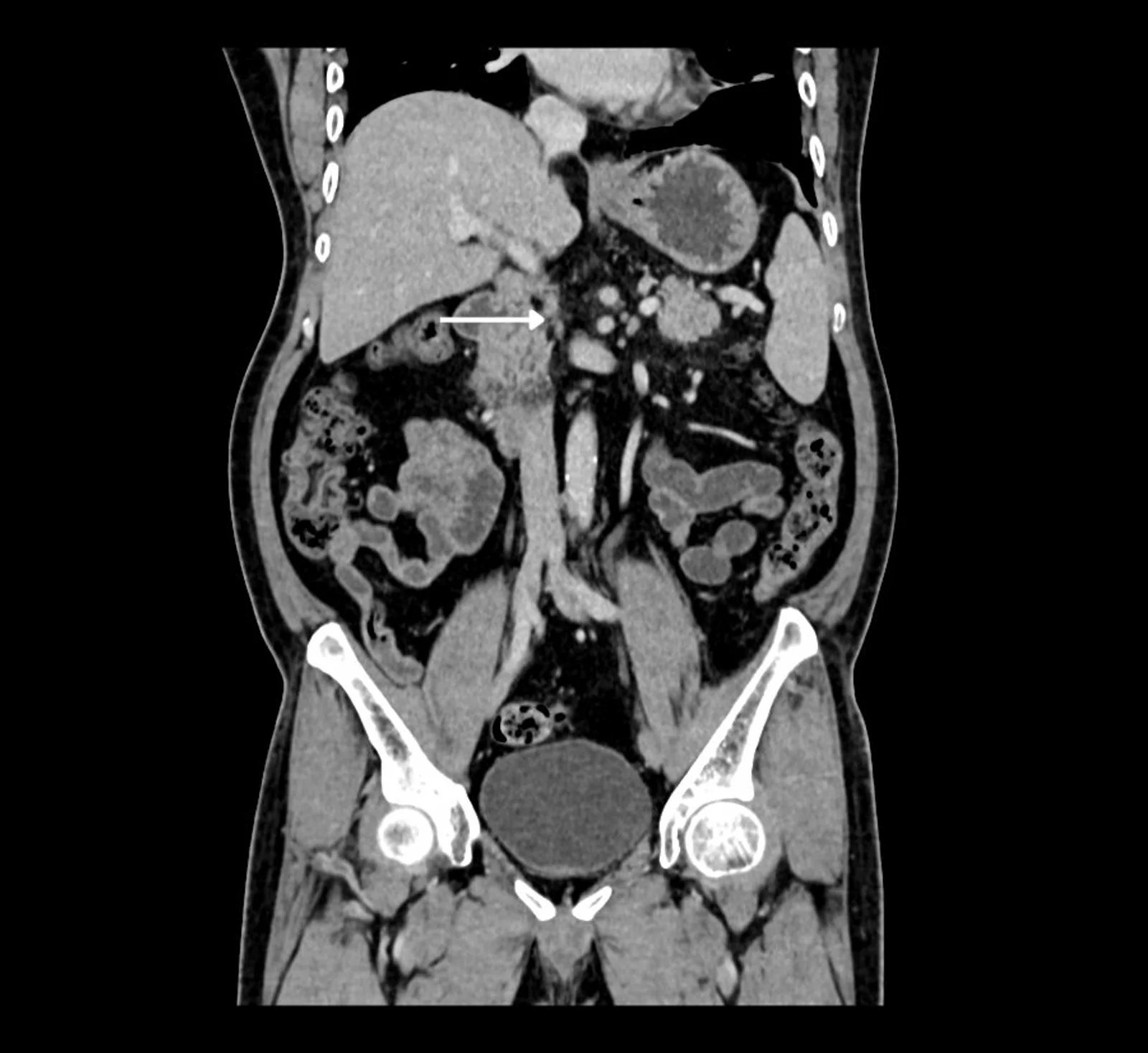
Coronal section of the contrast-enhanced computed tomography (CECT) abdomen showing distal tapering of the common bile duct (CBD) The arrowhead points toward the site of distal CBD tapering.

As the patient's symptoms persisted, contrast-enhanced computed tomography (CECT) of the abdomen was performed to exclude other intra-abdominal causes. The examination demonstrated a thick-walled gallbladder with cholelithiasis (Figures 2, 3), mild pericholecystic inflammatory changes, dilatation of the common bile duct with distal tapering (Figures 4, 5), and features of mild pancreatitis, although no discrete focal lesion was identified. 

**Figure 6:**
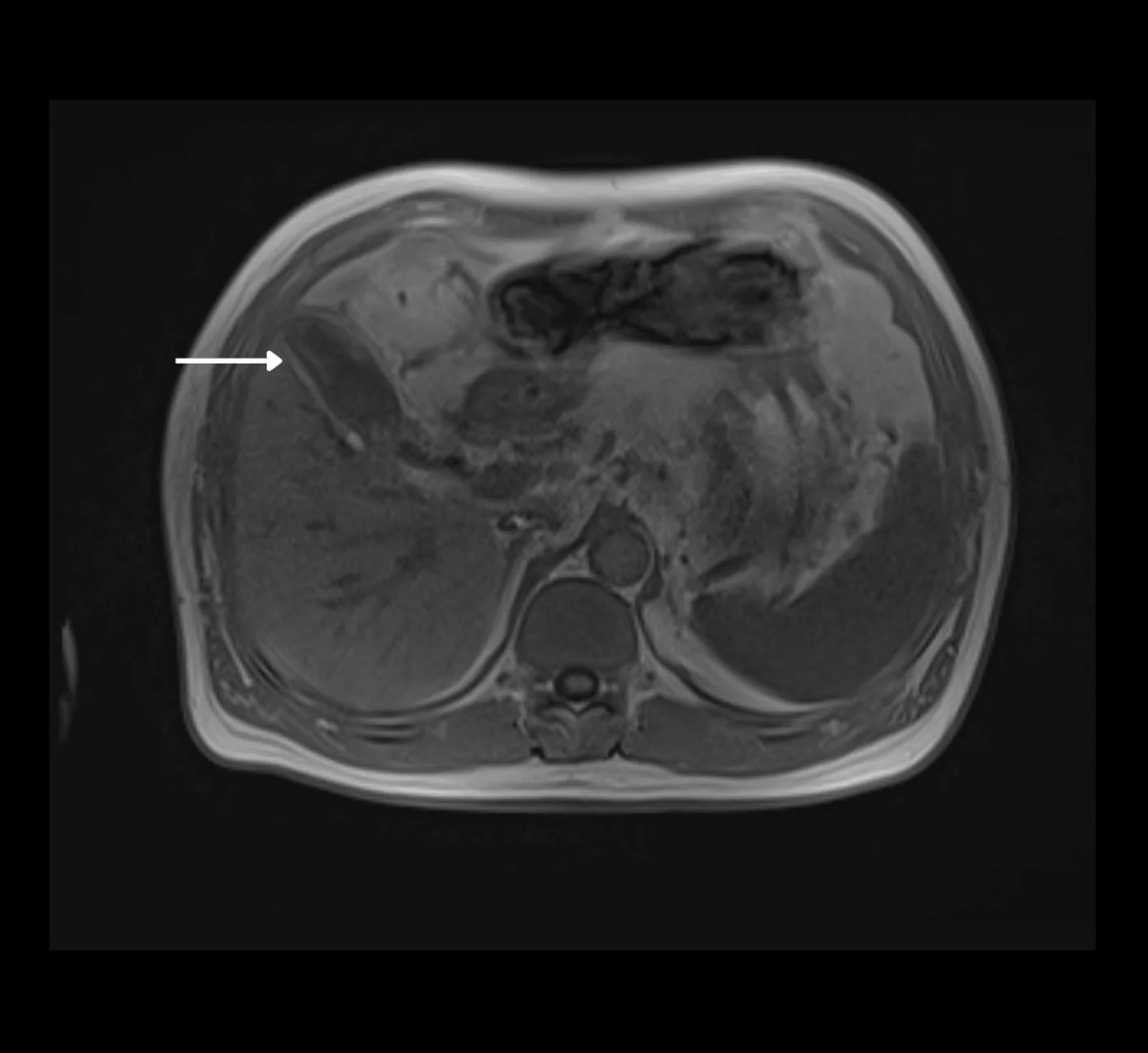
MRCP T1-weighted axial image showing focal asymmetric hypointense wall thickening involving the fundus and distal body of the gallbladder, measuring up to 6 mm The arrowhead points toward the focal wall thickening at the fundus.

**Figure 7:**
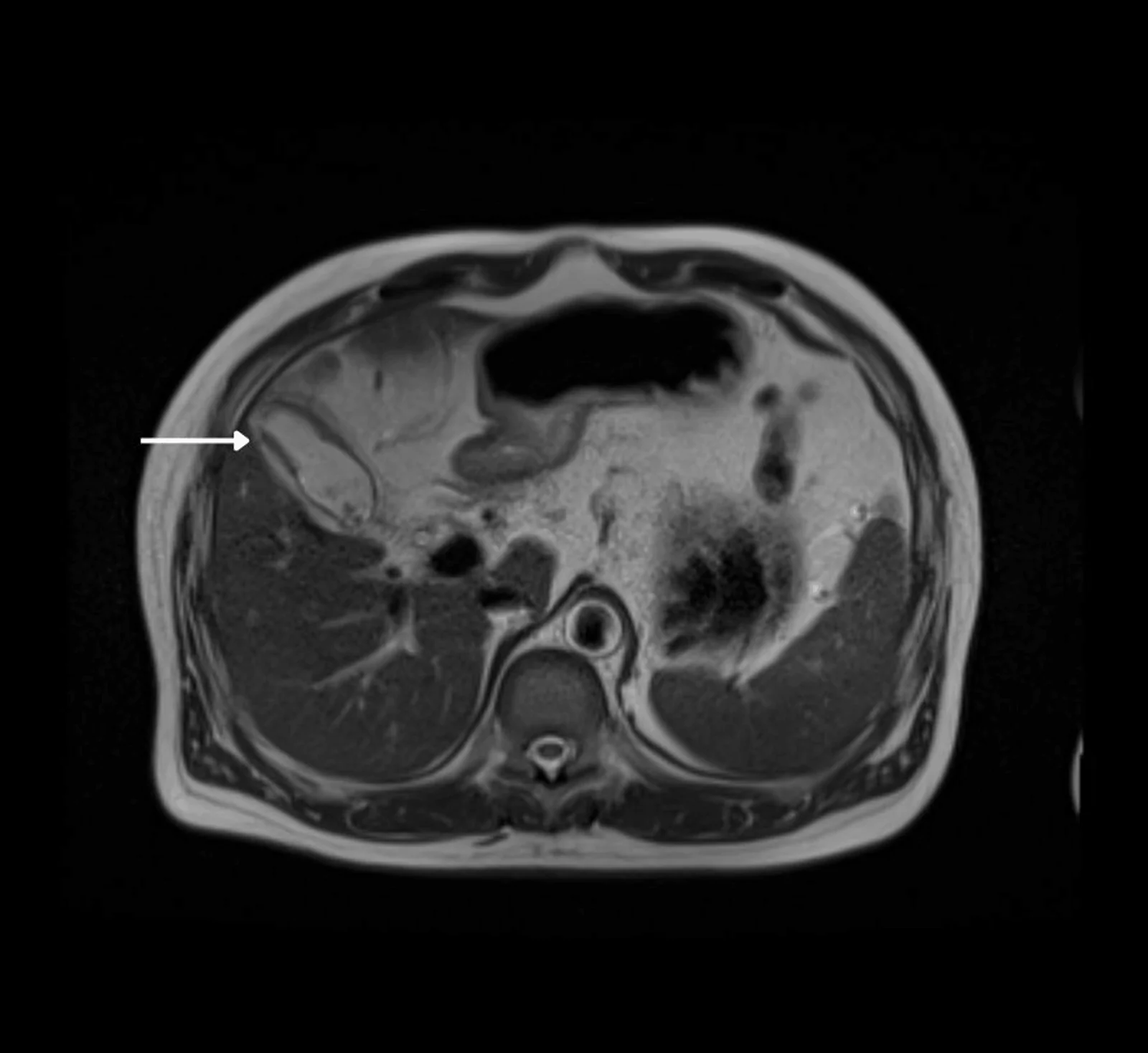
Magnetic resonance cholangiopancreatography (MRCP) T2-weighted axial image showing focal hyperintense wall thickening at the fundus of the gallbladder The arrowhead points toward the focal wall thickening at the fundus of the gallbladder.

**Figure 8:**
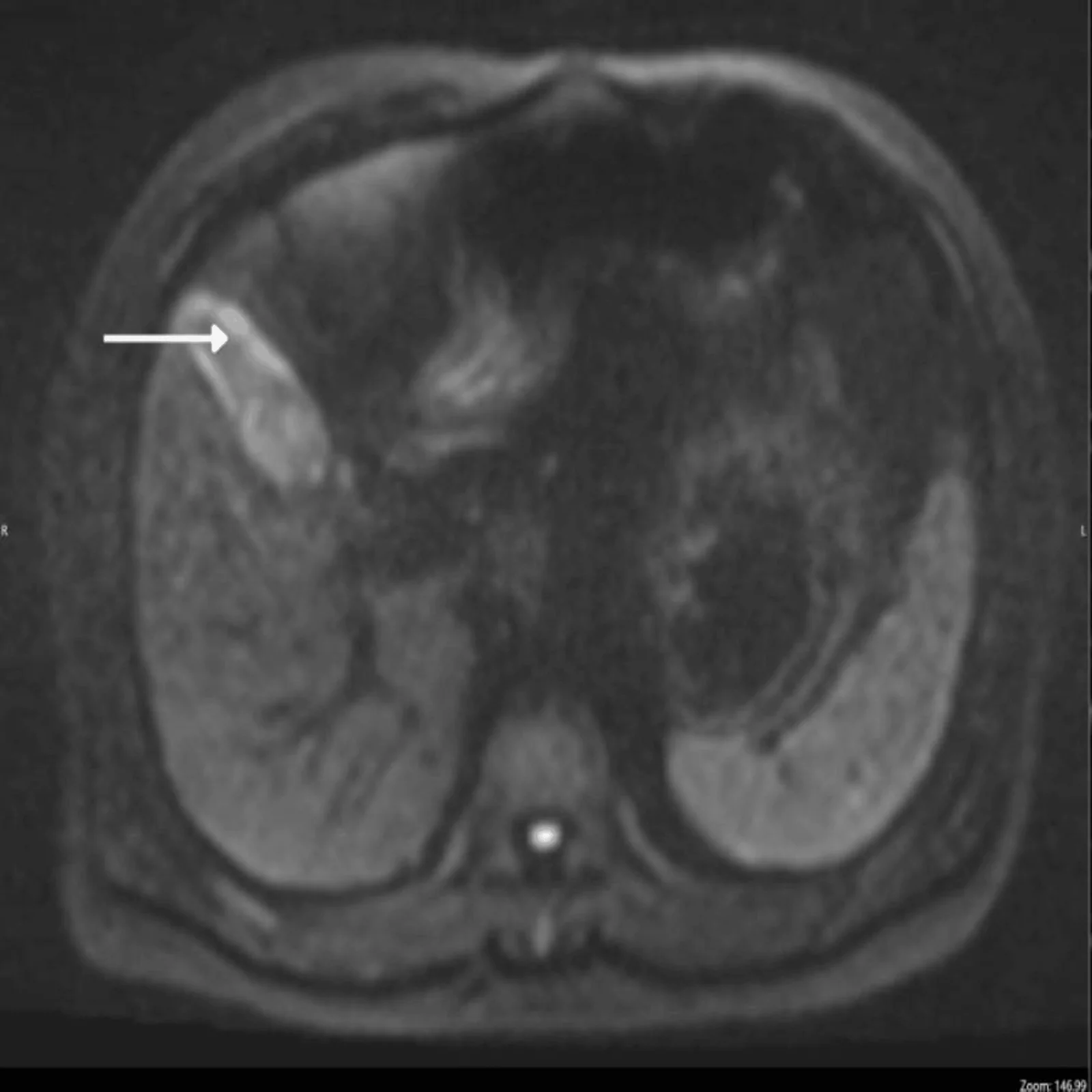
Axial diffusion-weighted imaging (DWI) image showing focal hyperintense wall thickening involving the fundus and distal body of the gallbladder, measuring up to approximately 6 mm The arrowhead points toward the focal wall thickening.

**Figure 9:**
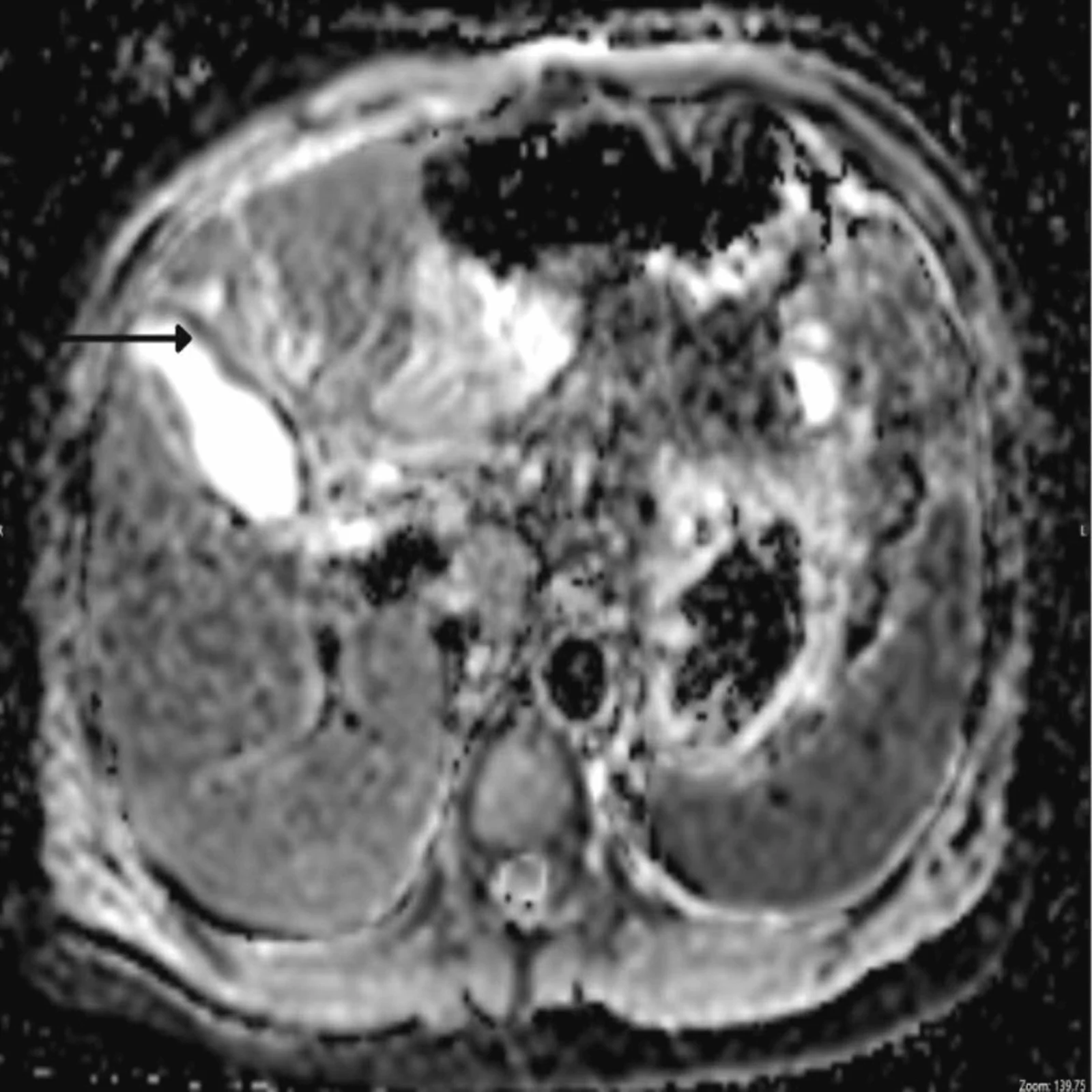
Axial apparent diffusion coefficient (ADC) showing focal hypointensity corresponding to wall thickening involving the fundus and distal body of the gallbladder, measuring up to approximately 6 mm The arrowhead points toward the focal wall thickening.

**Figure 10:**
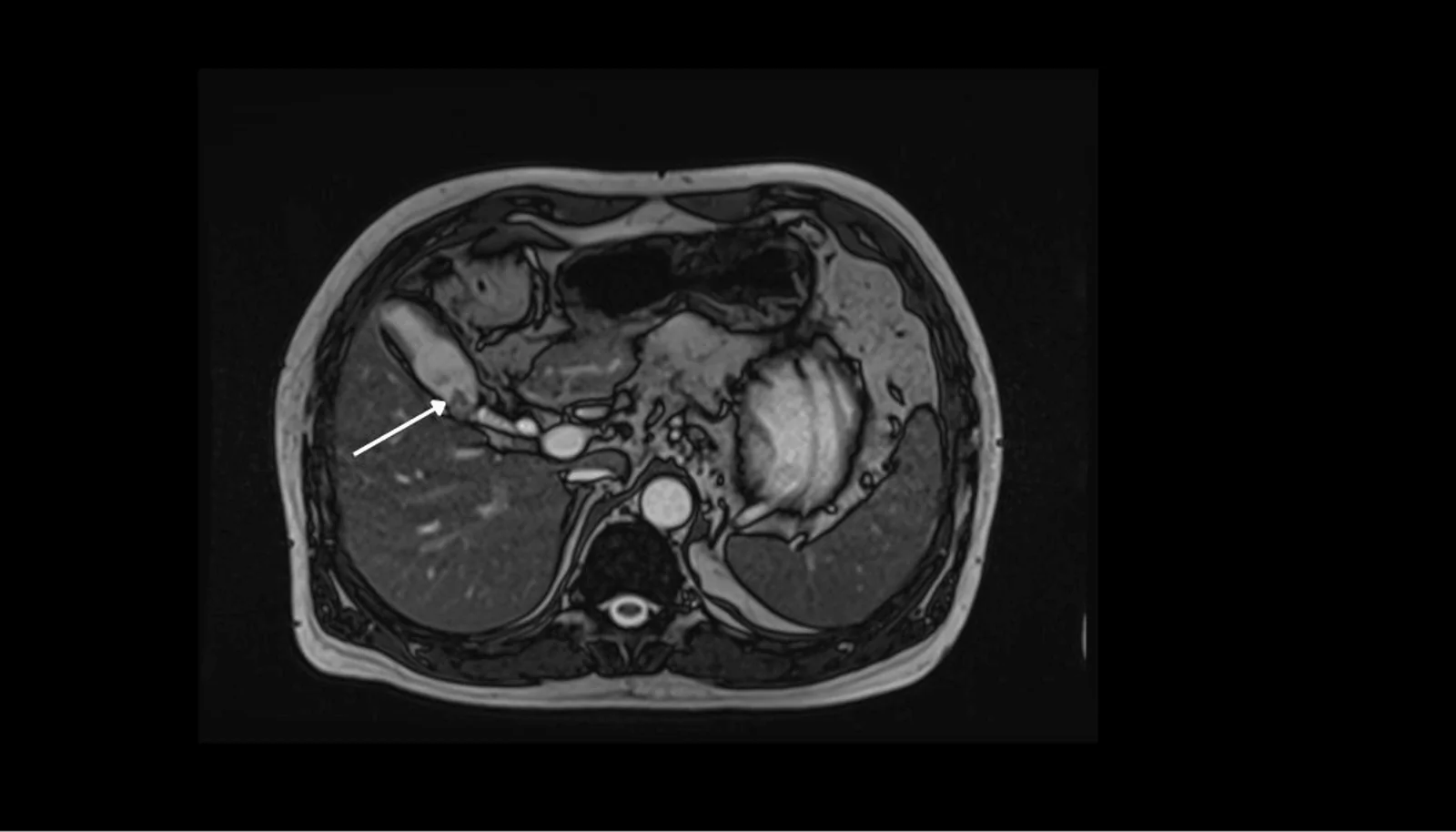
Magnetic resonance cholangiopancreatography (MRCP) T2-weighted axial image showing filling defects within the neck of the gallbladder measuring approximately 10 mm The arrowhead points toward the gallbladder neck.

Subsequent magnetic resonance cholangiopancreatography (MRCP) demonstrated focal asymmetric wall thickening involving the fundus and distal body of the gallbladder, measuring up to 6 mm in thickness (Figures 6, 7), with diffusion restriction demonstrated by hyperintensity on DWI and corresponding hypointensity on ADC (Figures 8, 9), and associated filling defects in the gallbladder neck measuring up to 10 mm (Figure 10). While the CECT findings favoured an inflammatory pathology, the focal asymmetric wall thickening with diffusion restriction on MRCP raised suspicion for gallbladder carcinoma.

These findings raised a radiological suspicion for gallbladder carcinoma. Although serum tumour markers remained within normal limits, the persistent focal wall thickening and diffusion restriction warranted surgical exploration. Table 2 shows the diagnostic imaging findings.

**Table 2:** Summary of diagnostic findings MRCP: magnetic resonance cholangiopancreatography, CEA: carcinoembryonic antigen, CA 19-9: carbohydrate antigen 19-9

Investigation	Principal findings	Diagnostic impression
Ultrasonography	Focal wall thickening involving the fundus and body of the gallbladder with cholelithiasis and echogenic intraluminal lesion/sludge; no definite sonographic features suggestive of malignancy	Further evaluation recommended
Contrast-enhanced CT abdomen	Thick-walled gallbladder with cholelithiasis, mild pericholecystic inflammatory changes, dilated common bile duct with distal tapering, mild pancreatitis; no discrete focal lesion identified	Findings favoured inflammatory pathology; malignancy could not be excluded
MRCP	Focal asymmetric wall thickening involving the fundus and distal body of the gallbladder (maximum thickness 6 mm) with diffusion restriction and filling defects in the gallbladder neck (up to 10 mm)	Suspicious for gallbladder carcinoma (radiological diagnosis: cT1N0Mx)
Serum tumour markers	CEA and CA 19-9 within normal limits	Biochemical findings not suggestive of malignancy
Intraoperative frozen section	No evidence of malignancy	Benign inflammatory pathology favoured
Final histopathology	Xanthogranulomatous cholecystitis without dysplasia or malignancy	Definitive diagnosis established

After multidisciplinary discussion, the patient was planned for laparoscopic cholecystectomy with intraoperative frozen section examination to guide the extent of surgical resection.

**Figure 11:**
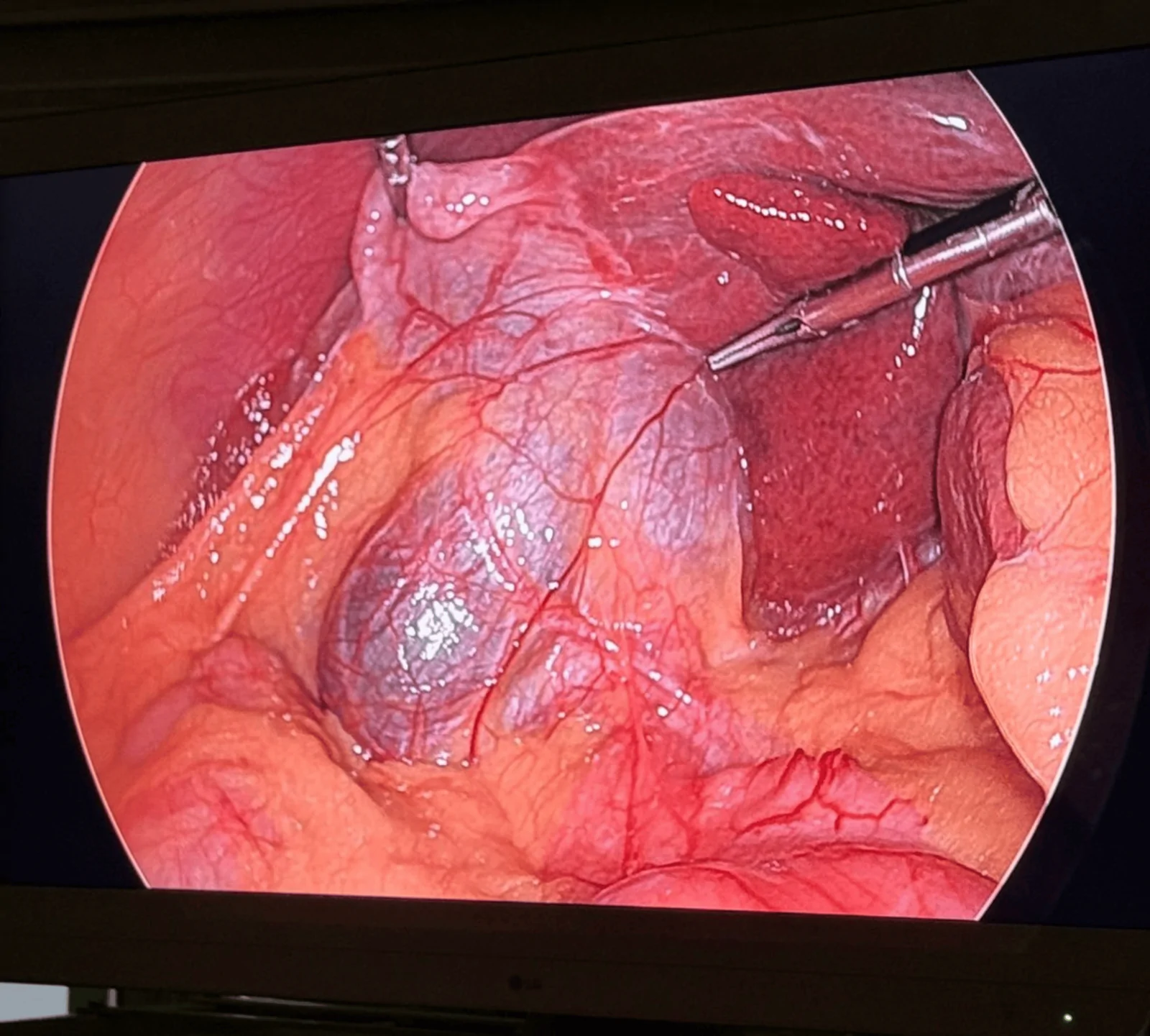
Intraoperative picture of flimsy omental adhesions to the gallbladder

Diagnostic laparoscopy revealed a thickened, oedematous gallbladder with flimsy omental adhesions involving Calot's triangle (Figure 11). The liver, stomach, small bowel, and large bowel appeared grossly normal. Careful dissection was performed above Rouviere's sulcus, and the cystic duct and cystic artery were identified. The critical view of safety (CVS) was achieved before clipping and division of both structures. The gallbladder was completely dissected from the liver bed, retrieved through the epigastric port, and submitted for intraoperative frozen section examination.

**Figure 12:**
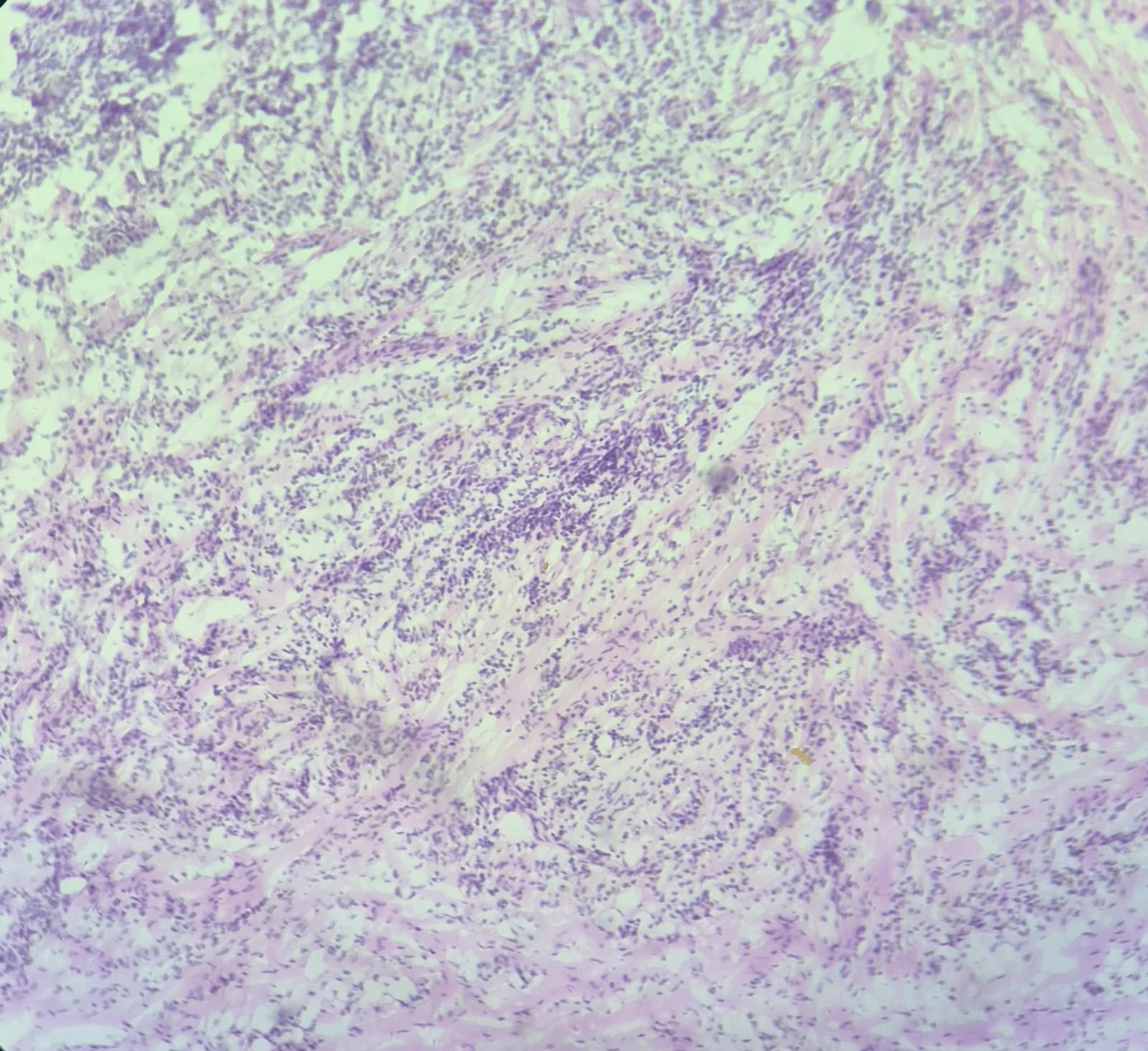
Intraoperative frozen-section examination revealing no evidence of malignant cells

Frozen-section examination of representative specimens obtained from both the anterior and posterior gallbladder walls demonstrated no evidence of malignancy (Figure 12), allowing completion of laparoscopic cholecystectomy without extending the procedure to radical resection. Haemostasis was secured, an 18-Fr suction drain was placed, and the operation was completed laparoscopically. The operative duration was approximately 90 minutes, with an estimated blood loss of 20 mL.

**Figure 13:**
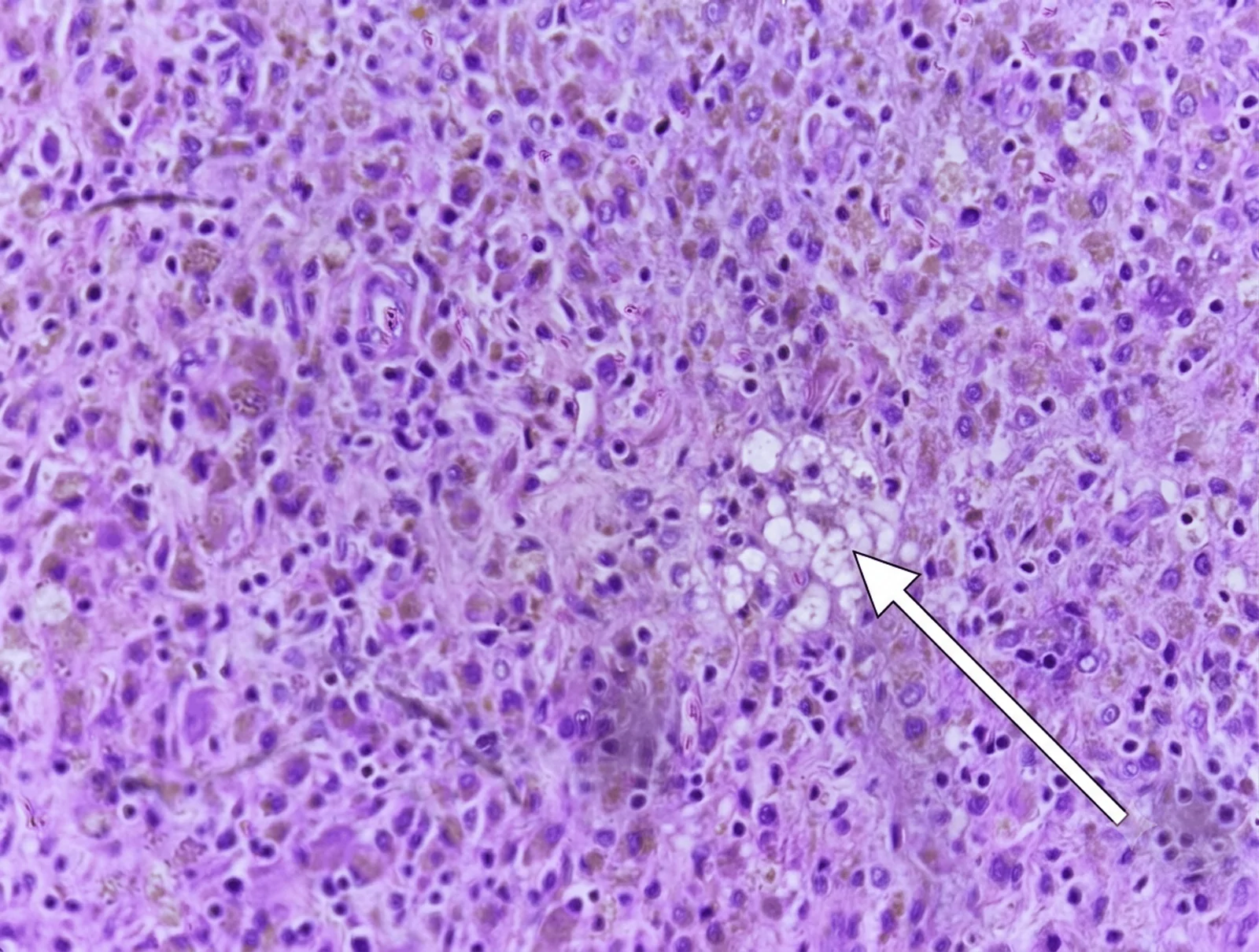
Final histopathological examination showing sheets of lipid-laden foamy macrophages (xanthoma cells), consistent with xanthogranulomatous cholecystitis. The arrowhead points to lipid-laden foamy macrophages (xanthoma cells).

**Figure 14:**
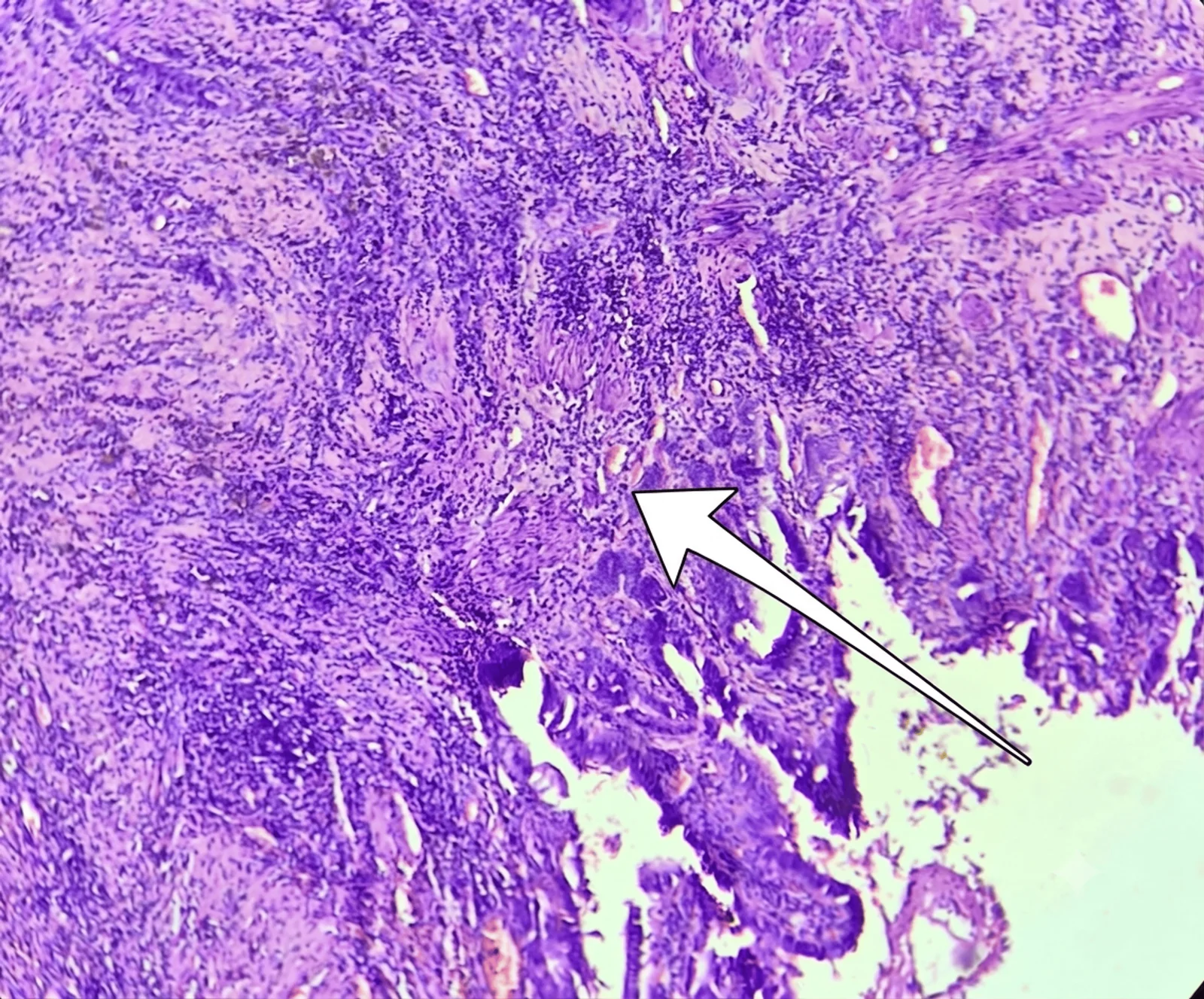
Histopathology image showing ulcerated gallbladder mucosa with dense mixed inflammatory infiltrates The arrowhead points toward inflammatory infiltrates.

**Figure 15:**
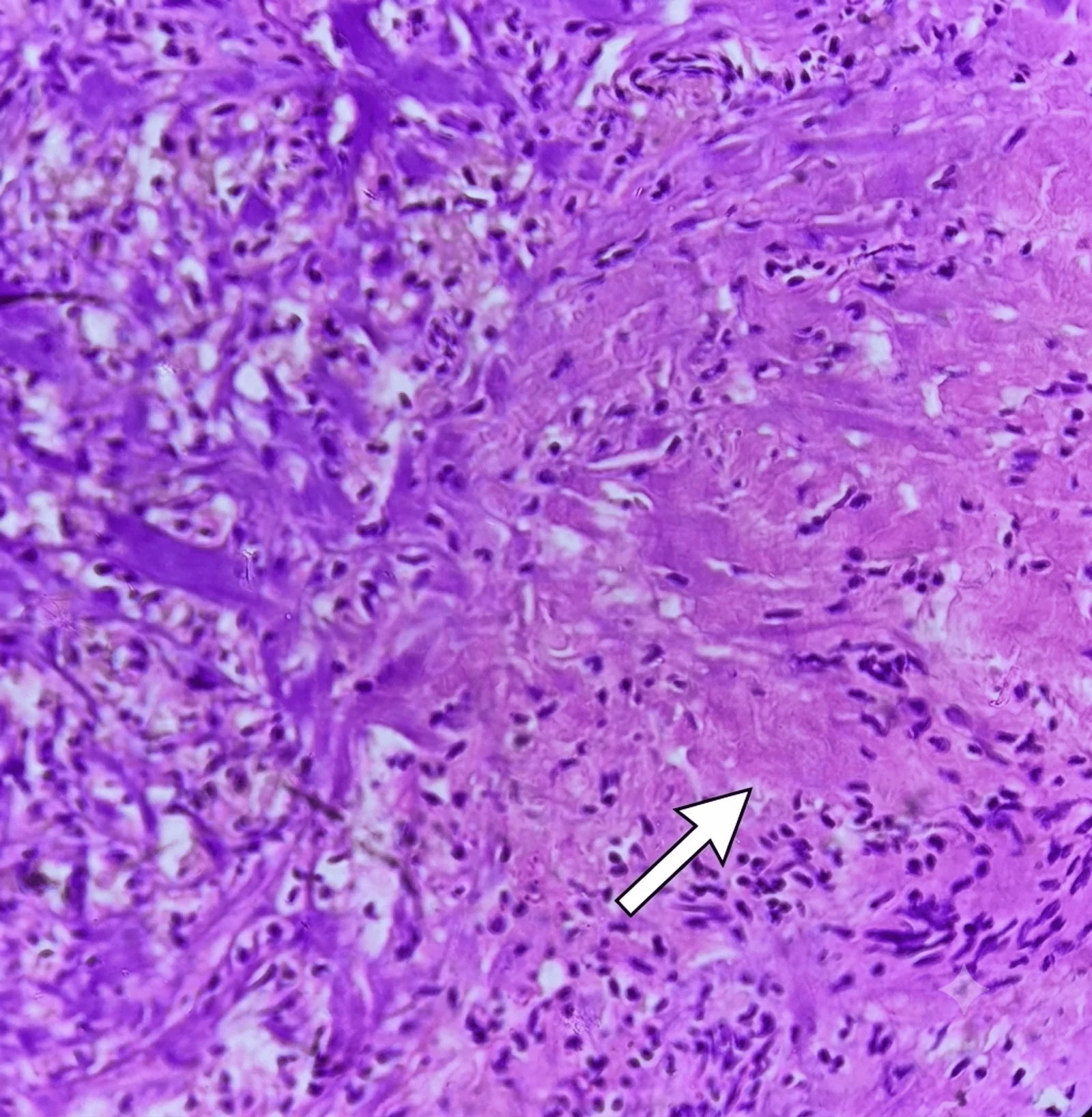
Histopathology image showing marked fibrosis and hyalinization The arrowhead points toward hyalinization.

**Figure 16:**
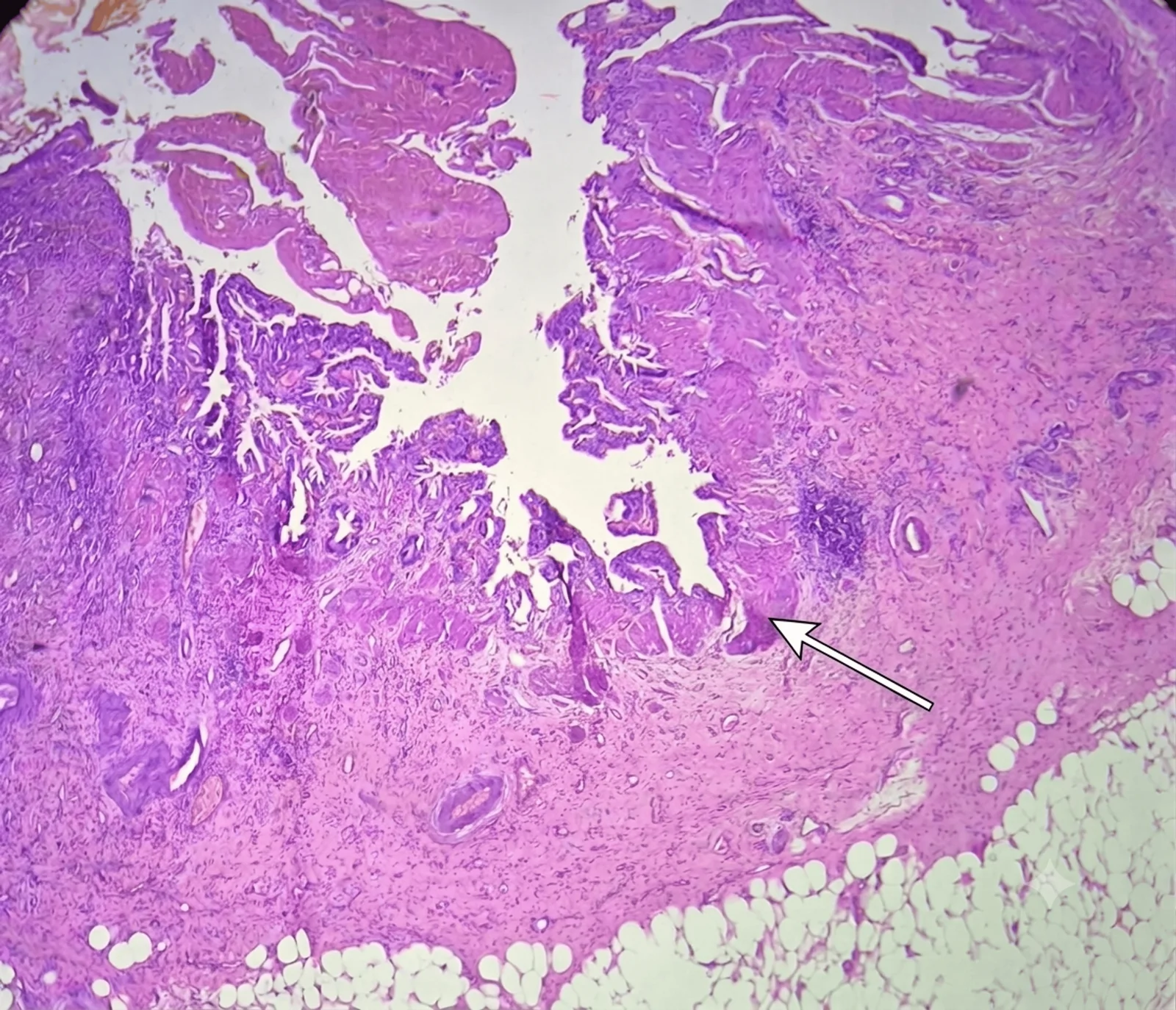
Histopathology image showing prominent Rokitansky–Aschoff sinuses. The arrowhead points toward Rokitansky-Aschoff sinuses.

Gross examination of the specimen revealed diffuse gallbladder wall thickening with focal grey-yellow areas and loss of the normal velvety mucosal architecture. Definitive histopathological examination demonstrated ulcerated gallbladder mucosa with dense mixed inflammatory infiltrates comprising lipid-laden histiocytes (Figure 13), multinucleated giant cells, lymphocytes, plasma cells, and neutrophils (Figure 14), together with marked fibrosis, hyalinization (Figure 15), congested blood vessels, and prominent Rokitansky-Aschoff sinuses (Figure 16). No epithelial dysplasia or malignancy was identified, confirming the diagnosis of xanthogranulomatous cholecystitis.

The postoperative recovery was uneventful. The nasogastric tube was removed on the day of surgery, while the abdominal drain and urinary catheter were removed on the first postoperative day. The patient tolerated oral feeds well, remained haemodynamically stable, and had satisfactory wound healing without postoperative complications. 

The patient was discharged on the sixth postoperative day in a stable clinical condition, with instructions to attend routine postoperative surgical follow-up and to continue regular follow-up with the Department of Gastroenterology for ongoing management of chronic hepatitis B infection (Table 3). 

**Table 3:** Timeline of clinical events USG: ultrasound imaging, CECT: contrast-enhanced computed tomography, MRCP: magnetic resonance cholangiopancreatography, XGC: xanthogranulomatous cholecystitis

Stage	Event
Presentation	Pain + fever + pruritus
Initial imaging	USG
Further evaluation	CECT
Advanced imaging	MRCP
Surgery	Laparoscopic cholecystectomy + frozen section
Histopathology	XGC
Outcome	Uneventful recovery

## Discussion

Preoperative differentiation between XGC and gallbladder carcinoma remains one of the greatest challenges in hepatobiliary surgery because both conditions frequently share overlapping clinical, biochemical, radiological, and intraoperative features. In a retrospective analysis of 8,213 cholecystectomy specimens, Şimşek et al. reported an incidence of only 0.91%, underscoring the rarity of XGC in routine surgical practice [5]. Despite its benign nature, the extensive fibro-inflammatory reaction associated with XGC produces marked gallbladder wall thickening, fibrosis, and inflammatory extension into adjacent tissues, frequently simulating locally advanced gallbladder carcinoma [6]. The present patient demonstrated this well-recognised diagnostic dilemma, presenting with upper abdominal pain, low-grade fever, generalized pruritus, mild weight loss, and progressive radiological abnormalities that initially raised concern for malignancy.

The diagnostic uncertainty evolved over the course of sequential imaging. Initial ultrasonography demonstrated focal wall thickening involving the fundus and body of the gallbladder with cholelithiasis but no definite sonographic features of malignancy. As symptoms persisted, CECT, performed to exclude other intra-abdominal pathology, demonstrated a thick-walled gallbladder with inflammatory changes and distal common bile duct tapering, but no discrete focal lesion, leaving the findings inconclusive. Subsequent MRCP demonstrated focal asymmetric wall thickening involving the fundus and distal body of the gallbladder, measuring up to 6 mm in thickness and showing diffusion restriction, significantly increasing radiological suspicion for gallbladder carcinoma. These findings are consistent with recent studies demonstrating that contemporary imaging modalities cannot reliably distinguish XGC from gallbladder carcinoma because both conditions commonly exhibit focal mural thickening, heterogeneous enhancement, diffusion restriction, and surrounding inflammatory changes [3,7]. Although serum carcinoembryonic antigen (CEA), carbohydrate antigen 19-9 (CA 19-9), alpha-fetoprotein (AFP)) were within normal limits in the present patient, normal tumour markers alone cannot reliably exclude gallbladder malignancy and should always be interpreted alongside imaging findings [7]. 

When diagnostic uncertainty persists despite comprehensive preoperative evaluation, intraoperative frozen-section examination becomes an important adjunct for determining the extent of surgical resection. In a large series of 575 patients undergoing surgery for suspected gallbladder malignancy, Patkar et al. reported that frozen section examination influenced intraoperative surgical strategy in 85.4% of cases, with a sensitivity of 88.3%, specificity of 99.6%, and an overall diagnostic accuracy of 95.1% [8]. The authors recommended frozen-section examination before undertaking radical resection whenever the diagnosis remains uncertain [8]. In the present case, frozen-section examination of representative samples from both the anterior and posterior gallbladder walls demonstrated no evidence of malignancy, allowing laparoscopic cholecystectomy to be completed without extending the operation to radical resection. Nevertheless, the frozen section should always be interpreted in conjunction with operative findings because sampling error and infiltrative tumour growth may occasionally limit diagnostic accuracy [8]. 

Laparoscopic cholecystectomy remains the preferred treatment for symptomatic XGC but may be technically demanding because of dense fibrosis, chronic inflammation, and distorted anatomy around Calot's triangle. Careful dissection and strict adherence to the principles of safe cholecystectomy are therefore essential to minimise the risk of bile duct injury [9]. In the present patient, a thickened, oedematous gallbladder with flimsy omental adhesions was encountered intraoperatively. Identification of Rouviere's sulcus and achievement of the Critical View of Safety enabled safe laparoscopic completion of the procedure despite a small cystic duct rent, which was immediately recognised and repaired without affecting the operative outcome. These findings are comparable to those reported by Torun et al., who demonstrated that laparoscopic cholecystectomy can be safely performed in most patients with XGC when meticulous surgical technique is employed, and conversion is reserved for cases with unsafe anatomy [6]. 

Definitive histopathological examination remains the gold standard for distinguishing XGC from gallbladder carcinoma. Characteristic microscopic findings include ulcerated gallbladder mucosa with lipid-laden histiocytes, multinucleated giant cells, lymphoplasmacytic infiltrates, fibrosis, and Rokitansky-Aschoff sinuses in the absence of epithelial dysplasia or invasive carcinoma [10]. Histopathological examination of the present specimen demonstrated all these classical features, confirming XGC and excluding malignancy. The marked fibrosis and chronic inflammatory infiltrates also explained the focal gallbladder wall thickening observed on preoperative imaging and the operative difficulty encountered during laparoscopic dissection [6,10]. 

This case emphasises that XGC should remain an important differential diagnosis in patients presenting with focal gallbladder wall thickening suspicious for malignancy. Sequential imaging may progressively increase suspicion for gallbladder carcinoma, but imaging findings alone are insufficient for definitive diagnosis. In carefully selected patients with indeterminate gallbladder lesions, normal tumour markers, selective use of intraoperative frozen section examination, meticulous operative technique, and definitive histopathological evaluation together facilitate accurate diagnosis while avoiding unnecessary radical surgery.

## Conclusions

XGC remains an important benign inflammatory condition that can closely mimic gallbladder carcinoma because of its overlapping radiological and intraoperative features. This case demonstrates that progressive imaging findings, despite negative tumour markers, may still raise a strong suspicion of malignancy, making definitive preoperative diagnosis difficult. In carefully selected patients with indeterminate gallbladder lesions, intraoperative frozen section examination can safely guide the extent of surgical resection, while definitive histopathological examination remains the gold standard for establishing the diagnosis. Recognition of this diagnostic entity and a systematic approach that integrates sequential imaging, meticulous operative technique, selective frozen-section examination, and histopathological confirmation can prevent unnecessary radical surgery and facilitate appropriate surgical management.
